# Impact of oral beta-blocker therapy on mortality after primary percutaneous coronary intervention for Killip class 1 myocardial infarction

**DOI:** 10.1007/s00380-015-0673-1

**Published:** 2015-04-12

**Authors:** Hirofumi Hioki, Hirohiko Motoki, Atsushi Izawa, Yuichirou Kashima, Takashi Miura, Souichirou Ebisawa, Takeshi Tomita, Yusuke Miyashita, Jun Koyama, Uichi Ikeda

**Affiliations:** Department of Cardiovascular Medicine, Shinshu University School of Medicine, 3-1-1 Asahi, Matsumoto, Nagano 390-0802 Japan

**Keywords:** Acute myocardial infarction, Beta-blocker, Primary percutaneous coronary intervention, Mortality

## Abstract

The use of beta-blockers therapy has been recommended to reduce mortality in patients with left ventricular dysfunction after acute myocardial infarction (AMI). Primary percutaneous coronary intervention (PCI), which has become the mainstay of treatment for AMI, is associated with a lower mortality than fibrinolysis. The benefits of beta-blockers after primary PCI in AMI patients without pump failure are unclear. We hypothesized that oral beta-blocker therapy after primary PCI might reduce the mortality in AMI patients without pump failure. The assessment of lipophilic vs. hydrophilic statin therapy in acute myocardial infarction (ALPS-AMI) study was a multi-center study that enrolled 508 AMI patients to compare the efficacy of hydrophilic and lipophilic statins in secondary prevention after myocardial infarction. We prospectively tracked cardiovascular events for 3 years in 444 ALPS-AMI patients (median age 66 years; 18.2 % women) who had Killip class 1 on admission and were discharged alive. The primary endpoint was all-cause mortality. The 3-year follow-up was completed in 413 patients (93.0 %). During this follow-up, 21 patients (4.7 %) died. In Kaplan–Meier analysis, patients on beta-blockers had a significantly lower incidence of all-cause mortality (2.7 vs. 7.3 %, log-rank *p* = 0.025). After adjusting for the calculated propensity score for using beta-blockers, their use remained an independent predictor of all-cause mortality (hazard ratio 0.309; 95 % confidence interval 0.116–0.824; *p* = 0.019). In the statin era, the use of beta-blocker therapy after primary PCI is associated with lower mortality in AMI patients with Killip class 1 on admission.

## Introduction

Beta-blockers have been established as important agents that reduce mortality and re-infarction in acute myocardial infarction (AMI) patients with left ventricular dysfunction [1–3]. In particular, the guidelines for management of ST-segment elevation myocardial infarction (STEMI) from the American Heart Association/American College of Cardiology and the European Society of Cardiology recommend the use of beta-blockers in AMI patients with heart failure or left ventricular dysfunction [4, 5]. However, these recommendations are based on the results of studies conducted in the fibrinolytic era [6]. Recently, primary percutaneous coronary intervention (PCI), which has been reported to be associated with a lower mortality rate than fibrinolysis [7], has become a mainstream treatment for AMI. Primary PCI also plays an important role in reducing the incidence of left ventricular dysfunction following AMI in many patients. Patients with Killip class 1 on admission have been reported to have better prognosis than patients with higher Killip classes [8].

The clinical benefit of beta-blocker therapy after primary PCI in patients without pump failure has not been well established. Clarifying the efficacy of beta-blocker therapy in reducing mortality might provide a novel therapeutic strategy for AMI patients who undergo primary PCI. Therefore, in the present study we investigated the clinical benefit of beta-blocker therapy after primary PCI for Killip class 1 AMI patients.

## Materials and methods

### Study population

This study was a retrospective sub-analysis of the assessment of lipophilic vs. hydrophilic statin therapy in acute myocardial infarction (ALPS-AMI) study, which included 508 patients enrolled between July 2008 and June 2010. The results of the ALPS-AMI study were described in detail in a recent publication [9]. In brief, the ALPS-AMI study [University Hospital Medical Information Network Clinical Trials Registry of Japan registration number (UMIN-ID) 000001521] was a prospective multi-center interventional study of AMI patients from 19 collaborating hospitals located in Japan. All patients enrolled in this study were randomly allocated to receive 10 mg of either atorvastatin or pravastatin once daily and followed up. Evidence-based use of other oral medications for AMI such as beta-blockers, anti-platelet agents, and renin–angiotensin–aldosterone system inhibitors was left to the treating physician’s discretion during the hospitalization period. The study protocol was developed in accordance with the Declaration of Helsinki and was approved by the ethics committee of each participating hospital. All patients gave written informed consent before participating in this study.

From the 508 patients registered for the ALPS-AMI study, we excluded patients who had Killip class ≥2 and individuals who died during hospitalization. We identified 444 patients who had Killip class 1 on admission and were discharged alive. The patients were retrospectively tracked for major adverse events from the time of enrollment. The primary endpoint of this study was all-cause mortality. We compared event rates in the patients who received oral beta-blockers (beta group) and those who did not (non-beta group).

### Definitions and procedures

As previously reported [10], myocardial infarction was defined as STEMI or non-ST-segment elevation myocardial infarction. Killip class 1 was defined as no evidence of heart failure on admission [11]. All patients were taken immediately to the cardiac catheterization laboratory for coronary angiography and PCI after receiving 200 mg of chewable aspirin and 300 mg of clopidogrel. The primary PCI was performed within 24 h after the onset of AMI, and heparin was given to achieve an activated clotting time between 300 and 350 s. Maintenance doses of 100 mg of aspirin and 75 mg of clopidogrel were used after primary PCI, as reported previously [12]. The final coronary blood flow after PCI was assessed according to the thrombolysis in myocardial infarction (TIMI) classification [13], with procedural success defined as TIMI flow grade of 3. Left ventricular ejection fraction (LVEF) was measured with echocardiography using the biplane Simpson’s method at the time of hospital discharge.

### Statistical analysis

Continuous variables are presented as mean ± standard deviation, whereas dichotomous variables are presented as numbers and percentages. Differences between patients on beta-blockers and those not on beta-blockers were compared using the Chi-squared test for categorical variables and the unpaired Student’s *t* test or the Wilcoxon rank-sum test, as appropriate, for continuous variables. The Kaplan–Meier test was used to analyze the effect of beta-blockers on all-cause mortality. The log-rank test was used to compare survival curves. Univariate Cox-proportional hazards analyses were performed to identify independent predictors of all-cause mortality. Effect modification between exposure to beta-blockers and other variables was investigated. A propensity score for receiving beta-blockers was incorporated into the models. The propensity score was calculated using a non-parsimonious multivariate logistic regression model in which the outcome variable was use of beta-blockers. From the variables that we could collect, we considered following as variables to potentially influence the prescription of beta-blockers; age, gender, LVEF at discharge, estimated glomerular filtration rate, a history of cerebrovascular disease, prior PCI, use of angiotensin-converting enzyme inhibitors or angiotensin receptor blockers, B-type natriuretic peptide level, and final TIMI grade ≤2. Covariate selection for model entry was based on clinical experience and identification of beta-blocker prescription. Appropriateness of the model was validated by Hosmer–Lemeshow goodness-of-fit test. The model which showed highest value in the Hosmer–Lemeshow goodness-of-fit test was estimated to be appropriate model. We calculated propensity score in several models using variables mentioned above and found the model including age, LVEF at discharge, estimated glomerular filtration rate, use of angiotensin-converting enzyme inhibitors or angiotensin receptor blockers, B-type natriuretic peptide level, and final TIMI grade ≤2, as an appropriate for evaluating the efficacy of beta-blockers based on the results of the Hosmer–Lemeshow goodness-of-fit test. Propensity score-adjusted multivariate Cox regression analysis was then performed. A *p* value <0.05 was considered to represent statistical significance. Statistical analysis was performed using the Statistical Package for Social Sciences, version 21 (SPSS Inc., Chicago, IL, USA).

## Results

### Baseline characteristics

The mean duration of follow-up was 1040 ± 186 days. The baseline characteristics of the study patients are shown in Table 1. Overall, the mean age was 65.2 ± 11.7 years, 81.8 % of the patients were men, and 81.8 % had STEMI. Of the traditional risk factors for atherosclerosis, approximately one-third of the patients had dyslipidemia, one-half had hypertension, one-quarter had diabetes mellitus, and two-thirds had a history of current or former smoking. PCI was successful in 86.9 % of cases. The mean LVEF at discharge was 56.0 ± 11.9 %, and 8.1 % of the patients had LVEF ≤ 40 %. Among the 444 patients, 251 (56.5 %) were prescribed beta-blockers after primary PCI, including carvedilol (92.8 %), bisoprolol (4.0 %), and others (3.2 %). Except for the history of previous coronary artery disease, infarct-related artery, and administration of angiotensin-converting enzyme inhibitors or angiotensin receptor blockers, no differences were observed between the 2 groups.

**Table 1:** Baseline characteristics stratified by beta-blocker therapy status

Variables	Beta group (*n* = 251)	Non-beta group (*n* = 193)	*p* value
Age (years)	65.2 ± 11.7	66.3 ± 11.5	0.327
Body mass index (kg/m^2^)	23.9 ± 3.2	23.9 ± 3.9	0.944
Female sex	50 (19.9 %)	31 (16.1 %)	0.179
Dyslipidemia	99 (39.4 %)	66 (34.2 %)	0.150
Low-density lipoprotein cholesterol (mg/dl)	133.1 ± 33.7	128.7 ± 34.7	0.198
High-density lipoprotein cholesterol (mg/dl)	47.0 ± 11.4	48.9 ± 12.0	0.115
Diabetes mellitus	61 (24.3 %)	46 (23.8 %)	0.500
Hemoglobin A_1C_ (%)	6.3 ± 1.3	6.3 ± 1.1	0.950
Hypertension	115 (45.8 %)	86 (44.6 %)	0.419
Smoking	168 (66.9 %)	121 (62.7 %)	0.204
Family history of coronary artery disease	60 (23.9 %)	38 (19.7 %)	0.172
Estimated glomerular filtration rate (ml/min)	71.8 ± 19.0	72.3 ± 19.9	0.805
Hemoglobin (g/dl)	14.5 ± 1.8	14.7 ± 2.9	0.437
B-type natriuretic peptide (pg/ml)	71.8 ± 19.0	72.3 ± 19.9	0.805
Left ventricular ejection fraction at discharge	56.0 ± 11.9	56.2 ± 10.6	0.796
Medical history			
Cerebrovascular disease	18 (7.2 %)	9 (4.7 %)	0.186
Prior percutaneous coronary intervention	16 (6.4 %)	22 (11.4 %)	0.045
ST-segment elevation myocardial infarction	206 (82.1 %)	157 (81.3 %)	0.363
Infarct-related artery			
Left anterior descending artery	133 (53.0 %)	73 (37.8 %)	0.001
Left circumflex artery	36 (14.3 %)	31 (16.1 %)	0.355
Right coronary artery	87 (34.7 %)	89 (46.1 %)	0.009
Multi-vessel disease	83 (33.1 %)	57 (29.5 %)	0.245
Final TIMI flow grade ≤2	36 (14.3 %)	22 (11.4 %)	0.221
Medications at discharge			
Statins	251 (100 %)	193 (100 %)	1.000
Angiotensin-converting enzyme inhibitors or angiotensin receptor blockers	228 (90.8 %)	150 (77.7 %)	<0.001
Calcium channel blockers	40 (15.9 %)	36 (18.7 %)	0.265

Data are shown as mean ± SD or as *n* (percentage)

*TIMI* thrombolysis in myocardial infarction

### Incidence of all-cause mortality

**Fig. 1:**
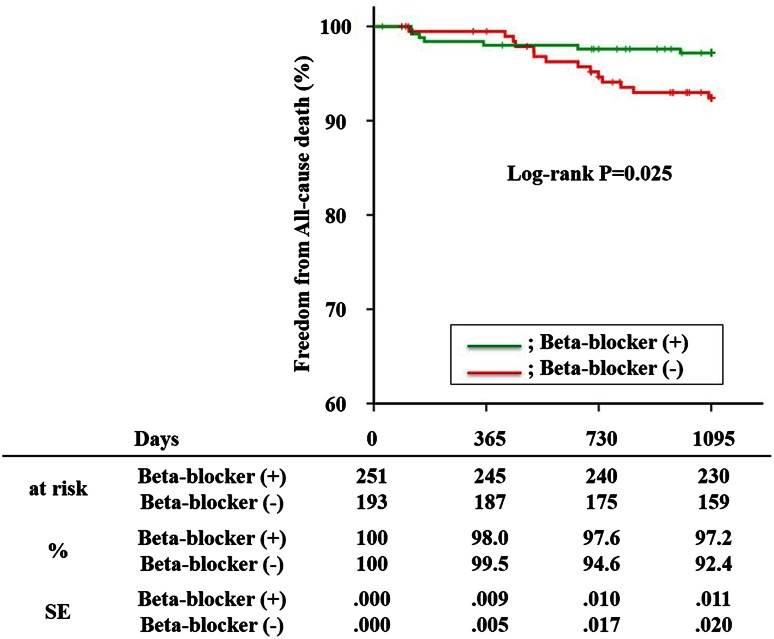
Kaplan–Meier analysis of all-cause mortality and beta-blocker therapy in the overall study population. During a mean follow-up of 3 years, there was a statistically significant difference in the incidence of all-cause mortality between the beta and non-beta groups (2.7 vs. 7.3 %, log-rank *p* = 0.025)

During the follow-up period, the cumulative incidence of all-cause mortality was 4.7 % (*n* = 21) for the entire study population, including 7 cases in the beta group and 14 cases in the non-beta group. Regarding the primary outcome, there were 5 cases of cardiac death due to heart failure and fatal arrhythmia, 9 cases of cerebrovascular death due to intracranial hemorrhage and stroke, and 7 cases of non-cardiovascular death. In the Kaplan–Meier analysis, the incidence of all-cause mortality was significantly lower in the beta group compared to the non-beta group (2.7 vs. 7.3 %, log-rank *p* = 0.025) (Fig. 1). The univariate Cox-proportional hazards analysis revealed that age [hazard ratio (HR) 1.057; 95 % confidence interval (CI) 1.013–1.103], estimated glomerular filtration rate on admission (HR 0.970; 95 % CI 0.947–0.993), and administration of beta-blockers (HR 0.367; 95 % CI 0.148–0.910) were significantly associated with lower all-cause mortality (Table 2). To minimize the influence of confounding variables, we also calculated the propensity score for receiving beta-blockers in this study population. The Hosmer–Lemeshow goodness-of-fit test for this model had a *p* value of 0.961. We then performed a multivariate Cox regression analysis with adjustment for the calculated propensity score, which also showed that beta-blocker therapy was an independent predictor of all-cause mortality (HR 0.309; 95 % CI 0.116–0.824; *p* = 0.019) (Table 2).

**Table 2:** Cox proportional regression analysis of all-cause mortality

Variable	HR	95 % CI	*p* value
Univariate Cox regression analysis			
Age	1.057	1.013–1.103	0.010
Female sex	0.463	0.108–1.989	0.301
Estimated glomerular filtration rate on admission	0.970	0.947–0.993	0.010
Angiotensin-converting enzyme inhibitors or angiotensin receptor blockers	0.464	0.180–1.197	0.112
Beta-blockers	0.367	0.148–0.910	0.031
Left ventricular ejection fraction at discharge	0.987	0.951–1.025	0.497
B-type natriuretic peptide	1.001	0.999–1.002	0.235
Final TIMI flow grade ≤ 2	2.176	0.797–5.939	0.129
Multivariate Cox regression analysis adjusted for propensity score^a^			
Beta-blockers	0.309	0.116–0.822	0.019

*HR* hazard ratio, *CI* confidential interval, *TIMI* thrombolysis in myocardial infarction

^a^The potential confounders used in the calculation of the propensity score included age, left ventricular ejection fraction, estimated glomerular filtration rate on admission, administration of angiotensin-converting enzyme inhibitors or angiotensin receptor blockers, B-type natriuretic peptide level, and final TIMI flow grade ≤2

## Discussion

In the present study, the use of beta-blocker therapy after primary PCI was significantly associated with a lower incidence of all-cause mortality in AMI patients with Killip class 1. Our results suggest that beta-blocker therapy may be beneficial for reducing mortality in relatively low-risk AMI patients after primary PCI.

The current guidelines on the use of beta-blocker therapy after AMI are based on the data obtained in the fibrinolytic era, and the clinical benefit of beta-blocker therapy was demonstrated only in patients with left ventricular dysfunction [1]. The guidelines for the management of AMI from the American Heart Association/American College of Cardiology and the European Society of Cardiology do not mention beta-blocker therapy after primary PCI. In the current era of primary PCI, it has been proposed that beta-blocker therapy might be considered in AMI patients according to their individual mortality risks [14]. It has also been reported that beta-blocker therapy was associated with reduced mortality in a subgroup of patients with low LVEF after primary PCI. Although AMI patients with Killip class 1 were shown to have a lower mortality than patients with higher Killip classes [8], the efficacy of beta-blocker therapy in reducing mortality was not fully characterized in such patients. Thus, our findings contribute new insights into the management of AMI patients.

The Harmonizing Outcomes with Revascularization and Stents in Acute Myocardial Infarction (HORIZONS-AMI) trial showed that failure to achieve the TIMI flow grade of 3 after primary PCI was a powerful predictor of mortality [15], and a final TIMI flow grade ≤2 after primary PCI for AMI was reported to be associated with poor outcomes [16]. In our study, the TIMI flow grade of 3 was achieved in 86.9 % of the patients, which is consistent with recent Japanese registry data [17]. Primary PCI delays the progression of ischemic myocardial injury and salvages the damaged but still viable myocardium [18]. Compared to fibrinolysis, primary PCI for AMI has been reported to reduce re-infarction and mortality, and it may also reduce the degree of left ventricular dysfunction after AMI. Although there were very few patients with left ventricular dysfunction in our study, patients receiving beta-blocker therapy after primary PCI had a significantly lower incidence of all-cause mortality compared to those who did not take beta-blockers. A recent study showed that beta-blocker therapy was associated with reduced all-cause mortality in AMI patients who survived for at least 30 days after discharge [19]. In addition to being consistent with this published result, our findings demonstrate for the first time that combining statin therapy with beta-blocker therapy may reduce the mortality in patients with no heart failure on admission.

As mentioned above, beta-blocker therapy after primary PCI was efficacious for reducing mortality in high-risk patients, but not in AMI patients overall [14, 20]. Bangalore et al. [21] reported the use of beta-blockers was not associated with a lower incidence of composite events in patients with prior MI. They also described that the beta-blocker use was associated with a lower incidence of cardiovascular events only in patients with recent MI (≤1 year). However, in the present study, a great reduction in mortality associated with the use of beta-blocker therapy was observed in patients at low risk of cardiovascular events. One difference between our study and the previous reports [14, 20, 21] was the proportion of patients on statins in the study population. Since in our case the original trial was designed for evaluating the efficacy of statins in AMI, all the subjects were on statins, compared to approximately 30–50 % of the patients in the previous reports. Although study of Bangalore et al. [21] was a large number, prospective cohort study, the proportion of primary PCI was not mentioned in prior MI cohort. Another previous study performed in the pre-primary PCI era demonstrated that the combination of beta-blockers and statins was associated with a reduction of the relative risk of all-cause mortality compared to the beta-blocker or statin therapy alone in myocardial infarction patients with heart failure [22]. This may explain why our data showed that beta-blockers were efficacious for reducing mortality in AMI patients without heart failure. Our findings suggest that the medical treatment after primary PCI should include beta-blocker therapy for all AMI patients, if possible. We propose that the combination of a beta-blocker and a statin may substantially reduce mortality in AMI patients without heart failure.

## Study limitations

Our study has several limitations. First, the sample size was relatively small. Thus, owing to the limited number of events, the study had low statistical power. Second, since the ALPS-AMI study was originally designed to assess the effect of statins in AMI patients, administration of beta-blockers after primary PCI was left to the treating physician’s discretion, which might have resulted in selection bias. To address this issue, we calculated the propensity score using potential confounders and performed a supplementary Cox regression analysis. Furthermore, no data were available on the doses or the way of titration of beta-blockers. The data about the onset-to-admission time, door-to-balloon time, duration of hospital stay, peak creatinine phosphokinase level after primary PCI, and initial TIMI flow grade were not assessed in this study. The day of discharge was not also assessed in this study. Therefore, the Day 0 was set as the day of admission, which might have influenced the results of the Cox-proportional hazard models in the present study. Finally, the mean follow-up duration was approximately 3 years, and long-term effects of beta-blockers after primary PCI should be investigated using a larger sample size and a longer follow-up period. Despite these limitations, we believe that the use of beta-blocker therapy after primary PCI might be associated with reduced mortality in AMI patients without pump failure in the current statin era.

## Conclusion

In the statin era, the use of beta-blocker therapy after primary PCI significantly reduces all-cause mortality compared to the treatment without beta-blocker therapy in AMI patients with Killip class 1. A further long-term follow-up study is required to evaluate the long-term effect of beta-blocker therapy on cardiovascular events after primary PCI.
